# Migration‐tracking integrated phylogeography supports long‐distance dispersal‐driven divergence for a migratory bird species in the Japanese archipelago

**DOI:** 10.1002/ece3.7387

**Published:** 2021-05-02

**Authors:** Daisuke Aoki, Haruna Sakamoto, Munehiro Kitazawa, Alexey P. Kryukov, Masaoki Takagi

**Affiliations:** ^1^ Department of Natural History Sciences Graduate School of Science Hokkaido University Sapporo Japan; ^2^ Frontiers in Environmental Sciences Graduate School of Agriculture Hokkaido University Sapporo Japan; ^3^ Laboratory of Evolutionary Zoology and Genetics Federal Scientific Center of the East Asia Terrestrial Biodiversity Far Eastern Branch of the Russian Academy of Sciences Vladivostok Russia; ^4^ Department of Natural History Sciences Faculty of Science Hokkaido University Sapporo Japan

**Keywords:** Brown Shrike, Japanese archipelago, light‐level geolocator, long‐distance dispersal, migratory route, species distribution modeling

## Abstract

Long‐distance dispersal (LDD) outside a species' breeding range contributes to genetic divergence. Previous phylogeographic studies of migratory bird species have not discriminated LDD from vicariant speciation in their diversification process. We conducted an integrative phylogeographic approach to test the LDD hypothesis, which predicts that a Japanese migratory bird subspecies diverged from a population in the coastal region of the East China Sea (CRECS) via LDD over the East China Sea (ECS). Haplotype networks of both mitochondrial and nuclear genes of its three subspecies were reconstructed to examine whether the Japanese subspecies of the Brown Shrike (*Lanius cristatus superciliosus*) diverged from an ancestral CRECS population. A species distribution model (SDM) for the Japanese subspecies was constructed using bioclimatic variables under the maximum entropy algorithm. It was projected backwards to the climate of the last glacial maximum (LGM) to infer the candidate source area of colonization. A migratory route of *L. c. superciliosus*, which possibly reflects a candidate past colonization route, was tracked by light‐level geolocators. Molecular phylogenetic networks suggest that the Japanese subspecies diverged from a population in the CRECS and maintained anciently diverged haplotypes. The SDM inferred that the emerged continental shelf of the ECS and the present CRECS were suitable breeding areas for the Japanese subspecies during the LGM. A major migratory route for *L. c. superciliosus* was inferred between the CRECS and the Japanese archipelago across the ECS. Our integrative approach supported the LDD hypothesis for divergence of the Japanese subspecies of the Brown Shrike. Shrinkage of the ECS may have been responsible for successful population establishment, due to a sufficient number of migrants overshooting to the Japanese archipelago from the CRECS. Our framework provides a new phylogeographic scenario for this region. Discriminating LDD and vicariance models helps improve our understanding of the phylogeographic histories of migratory species.

## Graphical abstract

We present a hypothetical phylogeographic scenario of divergence of a migratory bird species, the Brown Shrike in East Asia. A new scenario was proposed by integrative phylogeography including migration tracking and species distribution modeling. Long‐distance dispersal may have been important for its diversification process.

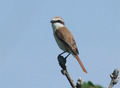

## 1 INTRODUCTION

How animal migratory behavior contributes to species distribution and speciation is a poorly studied area of biogeography (Greenberg & Marra, 2005; Winger et al., 2019). Evolutionary studies have recently detected greater opportunities for both population establishment in distant areas and genetic divergence in migratory than nonmigratory lineages (Lees & Gilroy, 2014; Rolland et al., 2014). Vagrancy, long‐distance dispersal (LDD) well outside a species' known range, is a candidate process for this evolutionary pattern. Vagrancy occurs when organisms in movement are influenced by wind or other meteorological conditions, and it derives mostly from migratory populations that have, for instance in the case of birds, drifted past or overshot their anticipated destinations during their seasonal migration (Newton, 2008). Therefore, vagrancy allows successful establishment of an allopatric breeding population (O'Connor, 1986; but see Lees & Gilroy, 2014) and its subsequent genetic divergence. Meanwhile, because migration‐related traits, such as a migration program forcing vagrants to continue migrating, may restrict establishment of a new population (Bensch, 1999), LDD‐driven divergence in these species could be condition‐dependent from the phylogeographical perspective. So far, for the divergence process of migratory species, many phylogeographic studies have assumed vicariance (e.g., Weir & Schluter, 2004; Zink et al., 2006), fragmentation of a once continuous breeding population by the appearance of a barrier to dispersal (Kropf et al., 2006). An alternative scenario, a disjunct breeding distribution, and divergence occur by LDD across a pre‐existing barrier, has never been tested. This is because migratory bird species are continental and widespread in their distribution (Somveille et al., 2018), thus the phylogeographic processes of their speciation cannot be empirically assigned to process (Albert et al., 2017). To understand the diversification process of migratory species, it is crucial to propose a phylogeographic scenario that would explain how and when migratory species diverge via LDD.

Owing to its insular nature, well‐documented paleogeography, and relatively high endemism of breeding migratory passerine lineages (Saitoh et al., 2015), the Japanese archipelago provides a suitable system within which to identify a biogeographic mode of speciation of a migratory lineage. During glacial periods of the Quaternary (approximately 2.7 million years ago to the present), due to lower sea levels, some parts of the Japanese archipelago were connected to the continent via land bridges, while other parts remained separated (Gallagher et al., 2015; Matsuzaki et al., 2019; Ohshima, 1990; Ota, 1998). Most terrestrial animal species currently occurring in Japan colonized the archipelago from the East Asian continent during glacial periods, and they subsequently diverged as Japanese endemics (McKay, 2012; Motokawa, 2017). Moreover, source continental populations could be either on the coastal region of the East China Sea (CRECS) or the northeast Asian continent (the Korean Peninsula, and northern China to Far East Russia), where distinct species or populations occupy their breeding ranges (e.g., Dong et al., 2015; Päckert et al., 2012; Saitoh et al., 2015; Zhao et al., 2017). This system, predicting how the Japanese archipelago was colonized (from which regional population and along which route), will help indicate which of the two following hypotheses are more plausible. (a) Colonization across more than 500 km wide East China Sea (ECS) from the CRECS beyond the normal dispersal distance of many migratory bird species (Paradis et al., 1998), implying LDD‐driven divergence; hereafter called the LDD Hypothesis. (b) Colonization by means of a range shift over a land bridge from the Korean Peninsula to Japan (where there is currently the Tsushima Strait), implies that the disappearance of the land bridge led to vicariant speciation; hereafter called the Vicariance Hypothesis. Moreover, different processes of population establishment and divergence are expected to result in different demography and different genetic signatures. Although molecular phylogenetics is one promising solution for distinguishing between these two hypotheses, it is often the case that postdivergence gene flow biases inference of species relationships and past demography (Leaché et al., 2014). The Japanese archipelago would provide a rare opportunity to discriminate the LDD and vicariance hypotheses, yet phylogenetic analyses only may not be sufficient for scenario testing.

We integrated a species distribution model (SDM) and migration tracking using light‐level geolocators with phylogeography. SDMs can infer suitable breeding areas of species during the last glacial maximum (LGM), ca. 20,000 years ago (20 kya) from present species distributions (Elith et al., 2011). Although predicted distributions during the LGM do not directly reflect the ancestral area of the Japanese population at the time divergence occurred, any continental range that was also suitable during one of several glacial periods may be interpreted as an area from which colonization occurred more likely than others (Zink & Gardner, 2017). An avian migratory route over a sea barrier between the Japanese archipelago and the Asian continent may be interpreted as a candidate for the past colonization route to the Japanese archipelago for the following reasons. First, an existing migration route over a large geographic barrier may reflect LDD in the past when the barrier was smaller (Newton, 2008; Winger et al., 2019). Second, the migratory route also partly retraces past shifts in the breeding range (Alvarado et al., 2014; Newton, 2008; Ruegg et al., 2006; Winger et al., 2019). Therefore, in this system, both modes of past colonization by a breeding population may be reflected in the migratory route over a present sea barrier.

**FIGURE 1:**
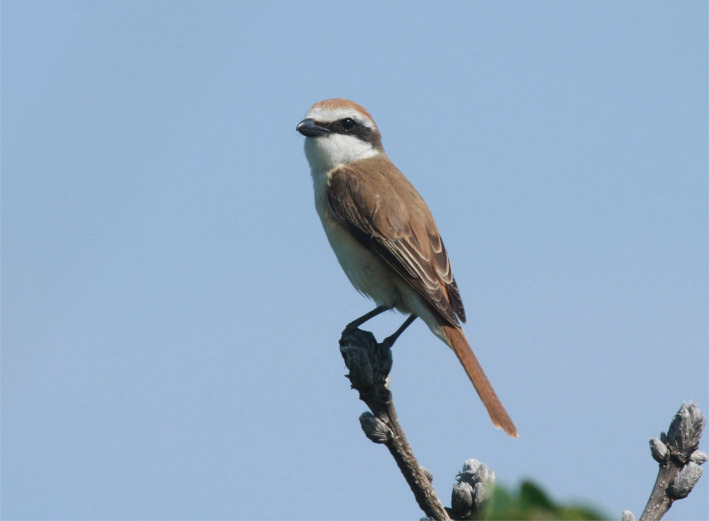
The Brown Shrike, *Lanius cristatus superciliosus* (Passeriformes, Laniidae)

**FIGURE 2:**
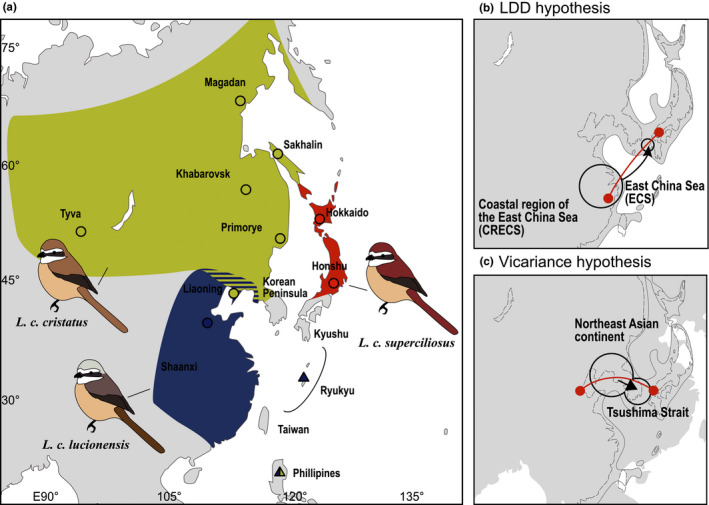
(a) DNA sampling localities. Circles and triangles represent samples from breeding and nonbreeding localities, respectively, and their colors indicate haplogroups inferred in the median‐joining haplotype network of COI (Figure 3a). Land colors indicate breeding ranges of presumed subspecies following Worfolk (2000). The striped region indicates a presumed hybrid zone between subspecies. Predicted colonization route (black arrows) and expected results from integrative approaches in (b) long‐distance dispersal (LDD) and (c) vicariance hypotheses for the Brown Shrike. Predicted migratory routes (lines with filled circles), and suitable glacial regions (open circles on the continent) under the two hypotheses are shown. Regions indicated in gray represent terrestrial areas during the Early to the Middle Pleistocene, and dotted lines indicate the present coastlines

We studied the Brown Shrike (*Lanius cristatus*), a long‐distance migratory passerine, as our first attempt to test these hypotheses because of the breeding distribution of its three subspecies. *Lanius cristatus superciliosus* breeds in the Japanese archipelago (Figure 1), *Lanius cristatus lucionensis* breeds in the CRECS, and *Lanius cristatus cristatus* breeds in the northeast Asian continent and most of Sakhalin Island (Figure 2a; Lefranc & Worfolk, 1997). The ancestral breeding area of this species has been inferred to be in the Oriental region (Fuchs et al., 2019), including the CRECS region. We sequenced two mitochondrial genes and two nuclear introns. We constructed their individual haplotype networks and a dated mitochondrial phylogenetic tree to detect genetic relationships among the three subspecies and genetic signatures related with past population demography. Results of the three components can be differently predicted from the two hypotheses in this framework. (a) The LDD hypothesis predicts that the archipelagic population has been derived either from the ancestral Brown Shrike population or *L. c. lucionensis* in the CRECS. Signatures of old genetic bottlenecks and genetic divergence may be expected because LDD and colonization should occur with a relatively small population. For the archipelagic population, the SDM predicts that CRECS was suitable during the glacial period, and a migratory route across the ECS between the Japanese archipelago and the CRECS is considered to have been likely (Figure 2b). (b) The vicariance hypothesis predicts that the archipelagic population genetically nests within, or is not well divergent from, the population on the northeast Asian continent, because the land bridge between the Korean Peninsula and the Japanese archipelago formed and disappeared repeatedly. For the archipelagic population, the SDM predicts that either current northern China or the Korean Peninsula were suitable during the glacial period, and a migratory route is expected to have existed over the Korean Peninsula from and to the Japanese archipelago (Figure 2c). Although each method individually involves limitations and provides weak support, in combination together they improve confidence in scenario selection for the colonization process of the Japanese migratory population. From the more plausible scenario based on our approach, we discuss the conditions under that LDD could have contributed to the genetic divergence of this migratory species.

## 2 MATERIALS AND METHODS

### 2.1 Study species and field procedure

To track the migratory routes of *L. c. superciliosus*, we captured 25 adult male shrikes in Hokkaido and marked them with leg rings. We attached MK5740 geolocator tags (BioTrack) with leg‐loop harness strings (Rappole & Tipton, 1991). We ensured that the total mass of the system (<1.1 g) did not exceed 5% of the birds' mass (Bridge et al., 2013). Eight birds (32%) returned to the breeding study site in 2018. Deployment of tags did not significantly change the return rate when compared with a previous report by Takagi (2003) (chi‐square test for independence; *χ* ^2^ = 0.11, *df* = 1, *p* = 0.74). Four males lost their tags before recapture. One male marked with leg rings disappeared before breeding in 2018 and did not return to the site in 2019, so it remained uncaptured (the presence of this bird's geolocator was not confirmed).

### 2.2 Laboratory procedure and haplotyping

We collected tissue and blood samples of *L. c. cristatus* from the Russian Far East; *L. c. superciliosus* from Hokkaido, and from Nagano Prefecture (Honshu Island); and *L. c. lucionensis* from the Ryukyu Islands. A total of 94 genetic samples were obtained. We also used database sequences of mitochondrial genes from GenBank and BOLD (Ratnasingham & Hebert, 2007) for coverage of its wider distribution (Figure 2a). Since the Ryukyu Islands and the Philippines are occupied only by nonbreeding (i.e., migrating and wintering) *L. c. lucionensis* (Lefranc & Worfolk, 1997; Severinghaus, 1996), and as this subspecies is easily separable from the other two subspecies based on morphological characteristics (Lefranc & Worfolk, 1997), samples from those locations were used as representatives of *L. c. lucionensis*. See Table S1.1 for details of samples and sequences.

Total genomic DNA was extracted from blood and tissue samples using DNeasy Blood & Tissue Kit (Qiagen). Two mitochondrial genes (complete cytochrome *b* (*cytb*), and partial cytochrome oxidase c subunit I (COI)) and two autosomal introns (myoglobin intron‐2 (MB), transforming growth factor beta 2 intron‐5 (TGFb2)) were amplified by polymerase chain reaction (PCR). The autosomal introns were sequenced only for a subset of the samples (Table S1.1). Automated sequencing was run on either an ABI7130 or ABI3130 Genetic Analyzer (ABI). Sequences were aligned using ProSeq v. 3.5 software (Filatov, 2009). To determine haplotypes of nuclear introns with multiple heterozygous sites, we used PHASE v. 2.1.1 (Stephens & Donnelly, 2003; Stephens et al., 2001). Haplotypes inferred with support values below 0.5 were not used in the following analyses. See Appendix S1 for details about primers, and PCR reactions.

### 2.3 Phylogenetic analyses

We constructed parsimony haplotype networks based on the median‐joining algorithm with postprocessing (using NETWORK 5.0.0.3; Bandelt et al., 1999; Polzin & Daneschmand, 2003) for each gene region with outgroup species (the Gray‐backed Shrike *Lanius tephronotus* and the Bull‐headed Shrike *Lanius bucephalus*). For the nuclear networks, we also included sequences from other Asian *Lanius* species available on the database because genetic structure was less apparent in these regions than in mitochondrial DNA (Table S1.1). We reconstructed mitochondrial phylogeny by using the two mitochondrial sequences. A dataset consisting of unique haplotypes of concatenated sequences of *cytb* and COI was prepared for tree reconstruction by the Bayesian inference (BI) method using BEAST v. 2.4.8 (Bouckaert et al., 2014). In the BEAST analysis, two genes were independently assigned to different partitions with the best substitution models determined by the lowest Bayesian Information Criterion on MEGA v. 7 (Kumar et al., 2016); HKY + G was selected for each gene (*α* = 0.10 for *cytb* and 0.19 for COI). Application of the standard avian clock rate for *cytb*, 0.0105/lineage/million years was justified since shrikes are passerines (Nabholz et al., 2016; Weir & Schluter, 2008). Markov chain Monte Carlo (MCMC) sampling proceeded for 50 million states with 100 thousand pre‐burn‐in chains. Convergence for each parameter was checked through Tracer v. 1.7.1 software (Rambaut et al., 2018). We followed Aoki et al. (2018) for other settings.

### 2.4 Species distribution modeling

We modeled the breeding range of *L. c. superciliosus* and projected it into the LGM. Data on occurrence in Japan were obtained from a summary of breeding localities reviewed by Kitazawa et al. (2020) (hereafter referred to as “review data”). Additionally, on 12 May 2019, we downloaded data from Global Biodiversity Information Facility (GBIF; https://www.gbif.org/, http://doi.org/10.15468/dl.igj5uw). A polygon bounding the presumed distribution of *L. c. superciliosus* according to Lefranc and Worfolk (1997) was used to clip the data. Records were limited only to the breeding season (May–August) to avoid integration of migrating birds. After a few quality‐checking processes, final coordinates were thinned to include only one occurrence per 20 km square covering the Japanese archipelago (Figure S1.1).

We constructed a model from the final coordinate data. Our purpose was to infer the continental area that might have been suitable for *L. c. superciliosus* as its breeding habitat during the LGM. Therefore, any ecological differences between the current archipelago and the continental area need to be integrated in the model (Elith et al., 2011). We clipped the environmental data to include the present distribution of the Brown Shrike regardless of subspecies and this environmental data were defined as the modeling domain. Of the available BIOCLIM variables (Booth et al., 2014) from WorldClim database (version 1.4; Hijmans et al., 2005), only one variable from each pair of correlated variables (|*r*| > 0.7) was used, resulting in seven variables: bio1, 2, 3, 5, 12, 14, and 15.

We used the R package “ENMeval” v. 0.3.0 (Muscarella et al., 2014) to execute the maximum entropy (MaxEnt) algorithm (Phillips et al., 2006) across a range of several settings. Combinations of three feature classes (linear, a combination of linear and quadratic, and hinge) and regularization multipliers ranging from 0.5 to 3 (increments of 0.5) were tested. The “maxent.jar” option was used as the model algorithm. From the geographical modeling domain defined above, 10,000 background points were sampled randomly. The “block” method was used to partition data for cross‐validation since it assesses model transferability better than other methods (Muscarella et al., 2014). The hinge feature, with a regularization multiplier of 1.5, was selected by the lowest AICc. The present distribution was predicted, and then it was projected to the LGM environmental data using cloglog transformation. The LGM environmental data were drawn by Hijmans et al. (2005) from three different climate models: the community climate system model (CCSM, Gent et al., 2011), the model for interdisciplinary research on climate model (MIROC‐ESM, Hasumi & Emori, 2004), and the Max‐Planck‐Inst. Für Meteorologie climate model (MPI‐ESM‐P, Liepert & Lo, 2013). Multivariate environmental similarity surface (MESS) was also calculated and extrapolation was restricted to regions indicated by negative values from this analysis (see Appendix S1 for the detailed procedures on the SDM analysis).

### 2.5 Migratory route analysis

Data retrieved from light‐level geolocators were analyzed in the R package “GeoLight” version 2.0.0 (Lisovski & Hahn, 2012) and “SGAT” version 0.1.3 (Wotherspoon et al., 2015). We prepared an R script by following the manual provided by Lisovski et al. (2019). We determined sunset and sunrise transitions for specific dates by light intensity thresholds of 0.5. We incorporated a statistical modeling approach, called the “group model” in a Bayesian framework implemented in “SGAT.” This model refines the estimated locations by means of three components: the twilight model, a movement model, and spatial masks. The twilight model incorporates the deviation between determined and actual twilight times in calibration periods of each tag. We used twilight data for 26 days after geolocator deployment as the calibration period. We fitted gamma distribution as an error distribution of detection of twilight. To define a movement model and spatial masks separately for stationary and movement periods, the ChangeLight function in “GeoLight” was applied to discriminate between these periods from the twilight data. A probability threshold for discrimination was set to 0.9. For the movement model, we set a flight speed prior with a gamma distribution for periods of movement (shape = 13, rate = 0.3), determined allowing for the average air speed of 12.9 m/s for the Red‐backed Shrike *Lanius collurio* (Bruderer & Boldt, 2001). We conducted a sensitivity analysis for the gamma distribution and found that priors did not greatly influence our interpretation of the results (Table S1.2). In the group model, twilight times grouped for a stationary period are treated as records at a single location, thus no movement model was defined. For a probability mask, birds must cross the sea between the Japanese archipelago and the continent and must stopover on land. Therefore, a spatial mask for stationary periods was designed only to include terrestrial land while both sea and land areas were allowed for movement periods. We modified our analysis slightly for bird #V5604‐025 because its geolocator stopped recording during the autumn equinox period (see Appendix S1 for details).

We interpolated latitudinal estimations around the equinox periods by setting a “tol” value between 0.08 and 0.1. As burn‐in and fine‐tuning, we ran 120,000 chains with three iterations, and the final paths were determined by the last 15,000 samples with three iterations. Regarding large uncertainties involved in migration estimations by geolocators (Lisovski et al., 2019), we standardized the posterior probabilities of location estimates on each raster cell, calculated their means among the three individuals, then presented them as a probability distribution in a region of interest where we expected birds to retrace the route of past colonization. We also inferred median paths of the three migratory tracks. Because birds spend more time at stopover sites than at an instantaneous point along a migratory path, the former are indicated with relatively higher probabilities than the latter.

## 3 RESULTS

### 3.1 Phylogenetic analyses

**FIGURE 3:**
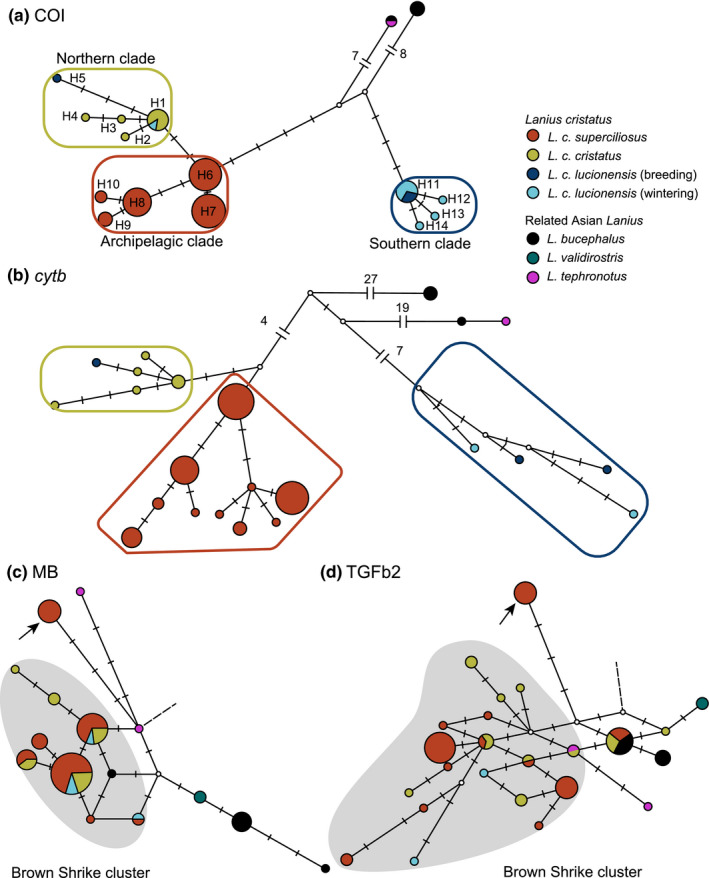
(a) A median‐joining haplotype network constructed using (a) 521 bp of the partial COI gene, (b) the complete cytochrome *b* (*cytb*), (c) the myoglobin intron‐2 (MB), and (d) trans growth factor beta 2 intron‐5 (TGFb2). Each circle indicates a unique haplotype, its size proportional to the number of samples in each network. Colors of filled circles indicate species or subspecies of samples used. One mutational gap is indicated by one bar, and a white node indicates a median vector. Clusters analogous to clades found in the Bayesian inference tree of concatenated mitochondrial genes (Figure S1.2) are indicated by colored quadrilaterals in the COI and *cytb* networks. Gray shading indicates clusters in which haplotypes of most Brown Shrikes samples were included, and arrows indicate highly divergent nuclear haplotypes. Nuclear haplotypes from genetically distant Asian *Lanius* species (*Lanius collurio* and *Lanius isabellinus* for both TGFb2 and MB, and *Lanius tigrinus* for MB only), which are not shown, are rooted to the haplotype or the median vector with broken lines

An MJ haplotype network of COI was constructed using 107 sequences from the species' range (Figure 3a). This network inferred three genetic groups, which were supported as distinct clades in the BI phylogenetic tree based on the concatenated mitochondrial sequences (Figure S1.2). The archipelagic, northern, and southern clades are thought to reflect the three extant subspecies (*L. c. superciliosus*, *L. c. cristatus*, and *L. c. lucionensis*, respectively; Figure 2a). In this network, a split between the southern and archipelagic clades was observed with nine mutational gaps. The archipelagic clade was inferred to be more ancestral than the northern clade. The major COI haplotype of the northern clade (H1) was shared among samples from a wide range of localities in Far East Russia (Table S1), and a star‐like pattern of this clade was found. COI haplotypes from two *L. c. lucionensis* samples (from Liaoning Province China and from the Philippines) were included in the northern clade. Although similar results were obtained from a haplotype network of *cytb* (Figure 3b) and the concatenated BI tree, they indicated the sister relationship between the northern and archipelagic clades. Genetic divergence among the three subspecies of the Brown Shrike was not apparent in either of the networks constructed for the two nuclear introns (Figure 3c,d). Meanwhile, one divergent haplotype unique to the archipelagic population was found outside a genetic cluster of the Brown Shrike in each nuclear region (arrows on Figure 3c,d). These haplotypes were neither shared with the other Brown Shrike populations nor included in the reticulation of the networks. They were as divergent as the haplotypes among other Asian *Lanius* species.

The BI tree indicated that the basal lineage of the Brown Shrike was the southern clade (Figure S1.2). The first split occurred between the southern and the archipelagic/northern clades, around 0.80 [0.55, 1.10] million years ago (Mya), corresponding to the late Early to Middle Pleistocene while the northern and the archipelagic clades diverged around 0.32 [0.20, 0.47] Mya, during the Middle Pleistocene (numbers within square brackets indicate 95% highest posterior density).

### 3.2 Species distribution modeling

**FIGURE 4:**
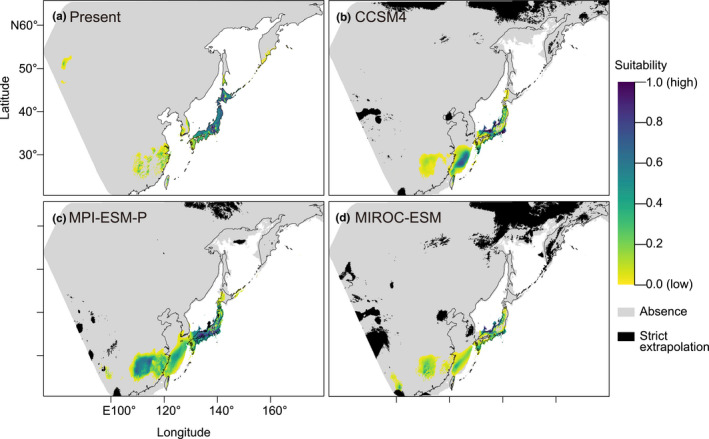
Estimated suitable breeding distribution for *Lanius cristatus superciliosus* during different time periods. A constructed model was projected to (a) the present climate, and (b–d) three different climate models for the Last Glacial Maximum (LGM, 20 kya). Suitability for a 2.5 min grid increases from yellow, green to indigo, whereas gray indicates absence. The black area is that of strict extrapolation indicated by multivariate environmental similarity surface analysis. Black lines indicate present coastlines. Note that the terrestrial landmass expanded during the LGM due to the lowered sea level

The present breeding distribution of *L. c. superciliosus* was modeled based on presence‐only data and seven noncorrelated bioclimatic variables using the MaxEnt algorithm (Figure 4). Minimum training presence omission rates for test localities were 1.9% and the model was evaluated to estimate suitable habitat without problematic overfitting. The present prediction of suitable areas (Figure 4a) reflected the taxon's known breeding range according to Lefranc and Worfolk (1997).

Projection of the distribution model to three climatic models of the LGM (Figure 4b–d) indicates that the coastal area of the currently submerged ECS continental shelf, and an inner continental range corresponding to the present CRECS, were suitable breeding habitat for *L. c. superciliosus*. The suitability of these regions was estimated to be relatively lower than in the Japanese archipelago, within which suitable breeding habitat receded southwards but largely remained during the LGM. Neither current Northern China, the Korean Peninsula, nor most of south‐westernmost Japan (the Ryukyu Islands) were predicted to be suitable for the species during the LGM, based on our model.

### 3.3 Migratory route analysis

**FIGURE 5:**
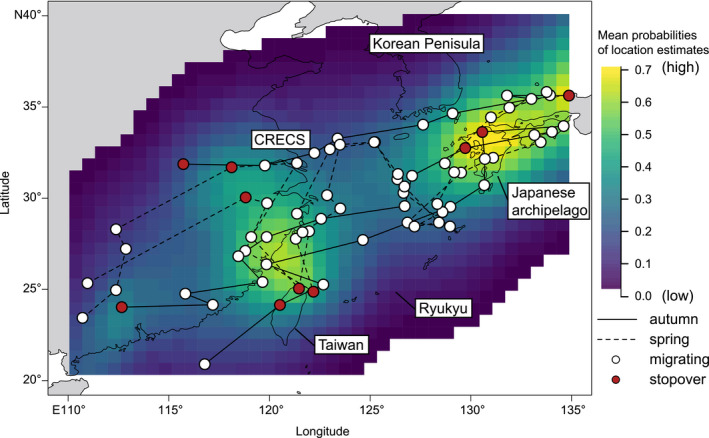
Migratory routes of three individual *Lanius cristatus superciliosus* around the East China Sea. Mean posterior probabilities of location estimates of the three birds for each raster cell are indicated by color gradation from yellow (high) to indigo (low). Circles connected by lines indicate median tracks of their inferred migratory routes (filled white circles indicate migrating and filled red circles indicate stationary). Solid lines indicate autumn migration and broken lines indicate spring migration. Labels indicate terrestrial landmasses (CRECS is coastal region of the East China Sea)

Under the Bayesian framework, autumn and spring migration routes were inferred for three individual *L. c. superciliosus* (Figures 5, S1.5). Full annual data were retrieved from two tags, and similarity was observed between their autumn and spring routes. One tag stopped recording on the continent due to cell failure 3 months after deployment. Despite uncertainties involved with location estimates, higher mean probabilities for migratory locations were observed among the three individuals both in the western part of the Japanese archipelago and along the CRECS to northern Taiwan. The probability map and the median migratory tracks suggest that birds migrated across the ECS between these two regions and did not detour over the Korean Peninsula. The data from the two successfully retrieved geolocators revealed that both shrikes had wintered in Indonesia, in the Sunda Islands, specifically on Java and Flores.

## 4 DISCUSSION

The archipelagic population of the Brown Shrike was inferred as a genetically divergent clade by the mitochondrial tree and networks. In the COI network, a phylogenetic split between the southern and archipelagic clades was inferred, while the northern clade was inferred to be a later‐derived clade (Figure 3a). Moreover, the archipelagic population preserved single haplotypes of the two nuclear introns, which were found outside the major Brown Shrike clusters and as divergent as those found from different Asian *Lanius* species (Figure 3c,d). These results agree with the LDD hypothesis that the Japanese population was once established from the ancestral population of this species and then isolated from the continental population for a long period. However, the sister relationship between the northern and archipelagic clades was inferred by the *cytb* network and the concatenated tree (Figures 3b, S1.2), which is possible under both hypotheses. Most of the nuclear haplotypes of the archipelagic population were shared with or genetically close to those of the continental populations of the Brown Shrike, also preventing confident scenario selection.

**FIGURE 6:**
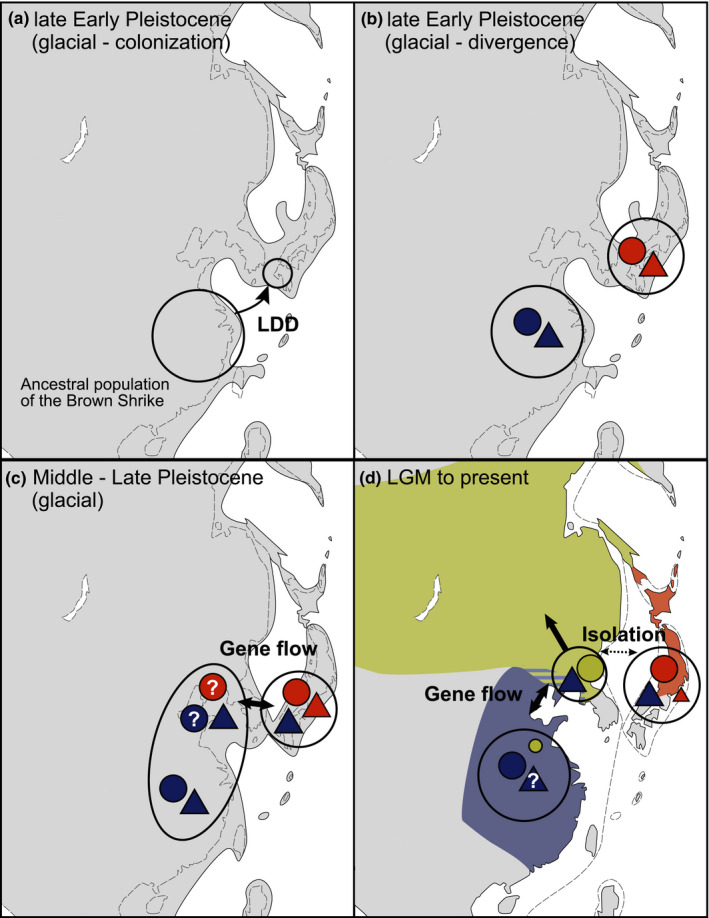
Schematic presentation of the hypothetical phylogeographic scenario of divergence in the Brown Shrike. Filled circles and triangles indicate mitochondrial and nuclear genes, respectively. Their colors correspond to presumable genetic groups, and their sizes correspond to the relative abundance in each population. Question marks indicate unconfirmed elements. (a) The ancestral population colonized the Japanese archipelago via LDD across the East China Sea (ECS) in a glacial period around the late Early Pleistocene and (b) genetically diverged subsequently. (c) In a later glacial period, gene flow occurred between the archipelagic and continental populations over a land bridge. (d) Although the origin of the northern population remains unclear, probably introgressed archipelagic mtDNA in the northern continental population diverged as the northern clade. Northward expansion of the northern continental population resulted in their present geographical distributions (land colors). The broken lines in each pane indicate the predicted coastlines in the previous inter/glacial periods, following Ota (1998)

Integration of the SDM and migration tracking were supportive of the LDD hypothesis. Suitable glacial breeding habitat for *L. c. superciliosus* was estimated to have existed around the CRECS during the LGM by the SDM (Figure 4), and its current migratory route was observed between the CRECS and the Japanese archipelago across the ECS (Figure 5). Within our scenario‐testing framework, our results indicate that the LDD‐driven divergence of a Japanese migratory subspecies across the ECS was more likely than the vicariance hypothesis. Signatures of old genetic divergence of the archipelagic haplotypes in both the COI and nuclear networks seem to reflect the same phylogeographic event (Zink & Barrowclough, 2008), possibly the ancient LDD‐driven colonization and divergence in the Japanese archipelago (Figure 6a,b). Therefore, less divergent nuclear haplotypes of the archipelagic population can be interpreted as the signature of postdivergence gene flow via secondary contact(s) between the continental and archipelagic populations (Figure 6c). Nuclear haplotypes introgressed from the continental population may have replaced most of the divergent haplotypes of the archipelagic population (Figure 6d). Possible postdivergence gene flow does not preclude our LDD‐driven divergence scenario of the archipelagic population. This is because the true population relationship is often underestimated by postdivergence gene flow (Leaché et al., 2014), and both climatic niches (Shaner et al., 2015) and migratory routes (Delmore & Irwin, 2014) should have been preserved despite postdivergence gene flow.

Our results cannot reflect gradual expansion of a population via normal dispersal over a terrestrial landscape. This is because, during the glacial periods around which divergence is estimated to have occurred, the gulf separating the Japanese archipelago, the Korean Peninsula, and the CRECS were relatively wide (Figure 6; Matsuzaki et al., 2019; Ota, 1998), compared with the LGM to which we projected our SDM. The rather ancestral and divergent mitochondrial and nuclear haplotypes preserved in the archipelagic population cannot be explained by this scenario either, because we expect gene flow, as extensive as the post‐divergence gene flow we inferred, impeding genetic divergence of the archipelagic haplotype. LDD colonization in a stepping‐stone manner along the Ryukyu Islands from the CRECS or Taiwan is also unlikely because our results predicted that most of the Ryukyu Islands were unsuitable as habitat during the LGM, and no migratory route was indicated along them, unlike the routes reported for other species (Koike et al., 2016; Shiu et al., 2006). Furthermore, since northern Taiwan was connected to the CRECS during the glacial period (Figure 6), the inferred migration aligns with our prediction.

### 4.1 Hypothetical phylogeographic scenario of the Brown Shrike

The ancestral breeding area of the Brown Shrike is believed to be in the Oriental region (Fuchs et al., 2019) where the CRECS is located. Given our LDD divergence scenario, the archipelagic population may have diverged from this ancestral population around the late Early Pleistocene. The ECS has remained open during a glacial period throughout the species' history. Meanwhile, the lower sea level led to the emergence of the continental shelf in the late Early to Middle Pleistocene (Matsuzaki et al., 2019; Ota, 1998). Our SDM analysis is in line with existing paleopalynological records indicating that the emergent continental shelf of the ECS was covered with grassland and sparse forest (Xu et al., 2010), which would have provided suitable breeding habitat for the Brown Shrike (Lefranc & Worfolk, 1997). Present vagrancy records of many migratory species in the Japanese archipelago during spring are probably due to overshooting (birds traveling too far because of wind and anticyclonic conditions; Newton, 2008) from the CRECS (Senzaki et al., 2019). A sufficient number of migrants toward the CRECS, therefore, may have accidentally traveled too far and arrived in the Japanese archipelago, resulting in a rare population establishment event by LDD (Figure 6a,b; Gillespie et al., 2012; MacArthur & Wilson, 1967). Increased proximity of the two landmasses during a glacial period may also have led sufficient propagule numbers to be carried to the Japanese archipelago to establish population by LDD since vagrancy to islands increases as the distance from the continental source area to the island decreases (Lees & Gilroy, 2014).

Meanwhile, the evolutionary origins of the two continental populations remain inconclusive. Genetically, the northern clade was found to be most distant from the southern clade but close to the archipelagic clade in our COI network, possibly suggesting divergence of the northern continental population from the archipelagic population by reverse colonization (Nishiumi & Kim, 2015). It is also possible that the northern clade became established via mitochondrial introgression from the archipelagic population to the ancestral continental population (Figure 6c), given our findings on post‐divergence gene flow between them. During the LGM, the present Korean peninsula and current northern China, with the Japanese archipelago connected to the continent at that time, were predicted to be suitable for both of the two continental subspecies (Figures S1.3, S1.4), supporting both possibilities. The small sample size of *L. c. lucionensis* and *L. c. cristatus* genetic materials contributes to our uncertainty of their evolutionary scenario. Whichever the scenario is, however, there should be ongoing gene flow between the two continental populations (Figure 6d), supported by the fact that the haplotypes of the northern clade found from *L. c. lucionensis*. The structure of the mitochondrial haplotype networks of the northern clade suggests that a small population located in the northern region of the continent during the glacial period, expanded its range, probably northwards, during the subsequent interglacial period (Avise et al., 1987). Disappearance of the land bridge and northward expansion of the northern clade could have resulted in isolation from the archipelagic population and the present distribution (Figure 6d). Although their past population dynamics and gene flow needs to be directly estimated by further genetic data and samples from wider regions to refine our hypothetical scenario, our integrative approach provides, at least, a new scenario for this region (Motokawa, 2017) and a better phylogeographic interpretation of the Brown Shrike.

### 4.2 Justification for the use of a migratory route as a proxy for the past colonization pathway

Our data on the migratory route of the Brown Shrike across the ECS is far too limited for population‐level inference, thus the universality of the route for the entire archipelagic subspecies remains unclear. Careful interpretation of our tracking data is required because individual variation in migratory routes is recognized (Stanley et al., 2012). Migratory routes inferred from the analysis also included large uncertainties (Figure 5) because of the methodological limitations of light‐level geolocators (Lisovski et al., 2019). However, Lefranc and Worfolk (1997) stated that, based on direct observation during migration, many *L. c. superciliosus* fly at least 700 km across the sea from Japan to the coast of China's Jiangsu Province or the Zhoushan Islands. Our geolocator data concur with their statement. Although further deployment of geolocators is needed, their statement guarantees that the focal sea crossing route is at least one of the major routes for the archipelagic population.

We have taken the recent view that idiosyncratic routes of obligate long‐distance migratory species are highly persistent traits throughout their evolutionary histories (Winger et al., 2019) in order to interpret part of our tracking data as the route of past colonization. However, the dominant view is to consider migratory routes as highly labile traits that are easily subjected to natural selection (Pulido, 2007; Winger et al., 2019). Thus, one could argue that the observed migratory route across the ECS is a result of optimization to the present environmental conditions and does not reflect past colonization (Alerstam, 2011; Berthold et al., 1992; Sutherland, 1998; Zink & Gardner, 2017).

For the following two reasons, we believe Winger et al.'s (2019) and our interpretation to be more plausible than the counterview. First, our system does not include any region that experienced severe glaciation. Earlier studies have examined species that inhabit regions previously covered by ice sheets during the LGM (Haché et al., 2017; Milá et al., 2006; Ruegg et al., 2006; Sokolovskis et al., 2018). Reverting from being migratory to becoming sedentary residents was probably a major response to glaciation, which was inferred by SDM analyses (Zink & Gardner, 2017). This reversion implies that migration is labile throughout a species' evolutionary history and migratory routes are likely to trace the postLGM expansion to the present breeding range. By contrast, we have shown that suitable breeding habitats for the archipelagic population remained in the Japanese archipelago even during the LGM (Figure 4b–d). This implies that the focal migratory route traces a distribution change before the LGM, supporting our view that idiosyncratic routes over a large barrier have been conserved. Second, similarity between autumn and spring migration across the ECS (Figure 5) may support the conservatism of the focal route. If a migratory route is optimized for the present environment, then different selection pressures upon passages in different seasons should independently shape the route (Stanley et al., 2012; Tøttrup et al., 2012). Generally, loop migration, in which spring and autumn migration routes are divergent, is thought to have evolved by retaining one ancestral route retracing colonization while deriving the other to adjust to current environmental conditions (Newton, 2008). Wind is an important determinant (Alerstam, 2001). This loop migration phenomenon has been shown for a population of the Oriental Honey Buzzard *Pernis ptilorhynchus* breeding in Japan. Its autumn migration involves crossing the ECS, whereas its spring migration route detours around the Korean Peninsula to cross a narrow sea channel to enter the Japanese archipelago (Nourani et al., 2016). It has been shown that a route over the Tsushima Strait was specifically suitable for spring wind conditions when wind directions and strengths over the ECS were unstable (Nourani et al., 2016; Yamaguchi et al., 2012). These conditions would be unfavorable also for passerines such as shrikes (Alerstam, 2001; Tøttrup et al., 2017).

### 4.3 Conclusion

Previous studies have illustrated a divergence scenario for migratory species that assumed vicariance in generating isolated populations (Hewitt, 2004; Weir & Schluter, 2004). While this scenario has provided suitable explanations for many cases, establishment of a new population from a distant area across a barrier as a result of LDD is likely to be their alternative explanation, given that migratory bird species have heightened opportunities for LDD and genetic divergence than nonmigratory lineages (Lees & Gilroy, 2014; Rolland et al., 2014). However, traditional frameworks based on the continental distribution of most migratory species have perhaps hindered the opportunities to test it (Warren et al., 2015). Our integrative approach using a Japanese migratory bird species has provided a suitable system within which to discriminate the mode of genetic divergence under a hypothesis‐testing framework. We have successfully provided a hypothetical scenario for how LDD and paleoenvironment have both contributed to the divergence of a migratory species. We have also shown that discriminating LDD from vicariance in our framework benefited a more comprehensive understanding of diversification mechanisms of migratory species in this region. We believe that consideration of LDD is also important for continental regions where most migratory species have diversified. Attention has recently been paid to LDD and colonization as an important phenomenon for distribution and speciation of continental migratory species (Rushing et al., 2015; Winkler et al., 2017). Further verification of the usability of our integrative approach among several species will facilitate our understanding of the relative importance of LDD to vicariance in the speciation of migratory birds and other animals.

## CONFLICT OF INTEREST

The authors declare no conflict of interest.

## AUTHOR CONTRIBUTIONS

**Daisuke Aoki:** Conceptualization (lead); data curation (lead); formal analysis (equal); investigation (equal); methodology (lead); resources (lead); visualization (lead). **Haruna Sakamoto:** Data curation (supporting). **Munehiro Kitazawa:** Conceptualization (supporting); investigation (supporting). **Alexey P. Kryukov:** Data curation (supporting); resources (supporting). **Masaoki Takagi:** Conceptualization (equal); data curation (supporting); formal analysis (supporting); funding acquisition (lead); investigation (supporting); methodology (supporting).

### OPEN RESEARCH BADGES

This article has been awarded Open Data, Open Materials, Preregistered Research Designs Badges. All materials and data are publicly accessible via the Open Science Framework at https://doi.org/10.5061/dryad.b5mkkwh8z and https://doi.org/10.5061/dryad.cfxpnvx2j

## Supporting information

Appendix S1

Figure S1.1

Figure S1.2

Figure S1.3

Figure S1.4

Figure S1.5

Table S1.1‐S1.2

## Data Availability

Genetic data is available at DDBJ (accession numbers are listed in Table S1.1). R scripts and the raw data for migratory route analyses and species distribution modeling are available at data repository (submitted to DRYAD with https://doi.org/10.5061/dryad.cfxpnvx2j).
