# Supplementary material for: Migration‐tracking integrated phylogeography supports long‐distance dispersal‐driven divergence for a migratory bird species in the Japanese archipelago
Source: Ecol Evol. 2021 May 2;11(11):6066–79. doi: 10.1002/ece3.7387 (PMC8207368; doi:10.1002/ece3.7387)
Supplement: Supplementary file 1 — Appendix S1 [file ECE3-11-6066-s005.docx]

**Migration-tracking integrated phylogeography supports a long-distance dispersal-driven divergence for a migratory bird species in the Japanese archipelago**

**AOKI Daisuke, SAKAMOTO Haruna, KITAZAWA Munehiro, KRYUKOV Alexey P & TAKAGI Masaoki**

**Appendix S1. Supplementary methods**

**Field procedure**

Field procedure was conducted with the utmost consideration of birds and their breeding. We limited periods of catching birds between late June and early August 2017 once their nestlings were more than seven days old so as to minimize disturbance to their breeding. Rest of seven days until fledging and the minimum period birds remained around their breeding territories after fledging (Lefranc & Worfolk, 1997) result in 26 days of calibration period. Natural tubular Teflon tape (Bally Ribbon Mills) was used as leg-loop harness strings since it prevents abrasion of birds’ bodies.

**Laboratory procedure**

The complete cytochrome *b* (*cytb*) gene and the partial cytochrome oxidase c subunit I (COI) gene were amplified with primer pairs newly designed for this study using Primer3Plus (Untergasser, Nijveen, Rao, & Bisseling, 2017); Lani_Cytb1+ (5’-CGGAAGGACTAGCAAACCTAC-3’) and Lani_Cytb2- (5’-GCTGGTGACAAAGGTTCGAC-3’) for *cytb* and Lani_COI+ (5’-CAACCCGGTGCTCTTCTAGG-3’) and Lani_COI- (5’-CCGAGGAACCCGATTGATAG- 3’) for *COI*. For nuclear introns, we used primers from previous studies (Heslewood et al., 1998; Primmer et al., 2002; Slade et al., 1993): Myo2 (5′GCCACCAAGCACAAGATCCC-3′) and Myo3F (5′-GCAAGGACCTTGATAATGACTT-3′) for myoglobin intron-2 (MB intron-2) and TGF5 (5′-GAAGCGTGCTCTAGATGCTG-3′) and TGF6 (5′-AGGCAGCAATTATCCTGCAC-3′) for transforming growth factor beta 2 (TGFb2) intron-5. Polymerase chain reactions (PCR) were performed in a final volume of 10 μL admixture containing 1 μL of template genomic DNA, 0.25 units of ExTaq (TaKaRa), 10 × Ex Taq Buffer (TaKaRa), 200 μM of dNTPs, 0.5 μM of each primer. PCR reactions were started with an initial cycle of 10 min denaturation, followed by 35 cycles of series of denaturation (30 s), annealing at 58.9 °C for COI, 52 °C for *cytb* (including 8 touch down cycles from 70 °C), 59.0 for MB intron-2 and 58.7 °C for TGFB2 intron-5 (30 s), and extension at 72 °C (1 min), terminated with 7 mins of a final cycle of extension. PCR products were purified by either the standard protocol using polyethylene glycol or ExoSAP-IT Express PCR Cleanup Reagents (Thermo Fisher). Cycle sequencing was performed with the purified PCR products using Big-Dye Terminator Cycle Sequencing kit v3.1 (ABI) with the corresponding primers used in PCR reactions and a pair of additional internal primers for *cytb* (Lani_cytb1-, 5’-TTGTTTGAGCCTGTTTCGTG-3’ as a reverse primer for the upper part and Lani_cytb2+, 5’-CAACCCGGTGCTCTTCTAGG-3’ as a forward primer for the lower part) which were also designed for this study with the protocol above.

**Species distribution modelling**

A polygon used to download GBIF data (bounded by 141.0 48.0, 137.9063 40.1224, 130.1727 34.1873, 131.8702 33.6877, 132.5 32.5, 140.3505 3.7972, 143.0 41.0, 150.0 45.0, 145.0 48.0, 141.0 48.0) was designed for the following purposes. Records from Kyushu Island from all the data sources were excluded by this polygon since breeding of this species is rare; *L. c. superciliosus* is a casual breeder only known in Oita prefecture (The Ornithological Society of Japan, 2012) while breeding records of *L. c. lucionensis* are only known as a migrant breeder around 1970’s (Imamura, 1976; The Ornithological Society of Japan, 2012). Only the southern part of Sakhalin island has a breeding record of *L. c. superciliosus* while the northern part of it is inhabited by *L. c. cristatus* (Lefranc & Worfolk, 1997). Therefore, we followed the distribution map by Lefranc & Worfolk (1997) to draw the polygon.

Because the review data from Kitazawa et al. (2020) is recorded by presence/absence information for approximately 20 km square grids tiled over the Japanese archipelago, we converted GBIF occurrence data to the same format and combined them with the review data. Centroids for the presence grids were treated as occurrence points for the following analysis, and hence the uncertainty of the coordinates is approximately 10 km around the points. This treatment also spatially filtered the occurrence dataset to reduce sampling bias. We checked that the GBIF records did not include any geospatial issues related to coordinate uncertainties or reprojection. Outliers were removed using the R package ‘CoordinateCleaner’ v. 2.0.11 (Zizka et al., 2019).

To define the extent of background samplings, we used the present distribution of the Brown Shrike regardless of the subspecies. Occurrence records of the other two subspecies of the Brown Shrike from GBIF were additionally downloaded, and a convex hull of aggregated occurrence records of the three subspecies with fixed distance buffer (1 degree) was used for clipping. Downloading information of the other two subspecies to draw an extent for background samplings is as followings: occurrences bounded by a polygon (132.0 40.2, 141.0 48.0, 145.0 48.0, 150.0 45.0, 180.0 59.0, 180.0 76.6, 81.0 76.6, 81.0 40.2, 132.0 40.2) were downloaded as data for *L. c. cristatus* on 12 May 2019 at https://doi.org/10.15468/dl.s5i70m, and those bounded by a polygon (98.0 23.28, 118.64 23.15, 127.56 31.54, 132.0 40.2, 81.0 40.2, 98.0 23.28) were downloaded for *L. c. lucionensis* on 12 May 2019 at https://doi.org/10.15468/dl.2qumce. To exclude occurrences of migrating birds, only data from June to August and June to July were selected for *L. c. cristatus* and *L. c. lucionensis*, respectively.

For a graphical presentation of the results, we used the R package ‘dismo’ (Hijmans, Phillips, Leathwick, & Elith, 2013) to cut values lower than a threshold determined by ‘prevalence’ (which finds a value closest to the observed prevalence) for graphical presentation.

We also constructed SDMs for *L. c. cristatus* and *L. c. lucionensis* and projected them to the three different climate models of the last glacial maximum (LGM). We thinned the occurrence data of the two subspecies downloaded above to retain the maximum number of records, all of which are at least 40 km apart. We constructed models with these thinned records and the same bioclimatic variables used for the model in the main text. Projections to the LGM were only presented in FigureS1.3 and S1.4.

**Modification on migratory route analysis for a bird with tag #5604-025**

#V5604-025 was prepared differently from the method described above because its geolocator stopped recording during the autumn equinox period. The raw data showed a gradual increase in uncertainty of location estimate closer to the equinox, but it seemed certain that the bird had migrated in a southerly direction through the Japanese archipelago. However, by including twilight data around the equinox and applying the same spatial mask as the other two individuals, the large continental area acted as a magnet and the bird ended up migrating northward to Mongolia which is not biologically plausible. Since data around the equinox have large uncertainties in latitudinal estimates, the continental area could easily attract the migratory path. Therefore, we constructed a separate spatial mask by taking a product of the original mask and the combined posterior probabilities of the annual presence of the other two birds. We also omitted twilights after 16^th^ of September in 2017 (a week before the equinox) because we cannot afford the benefit of interpolation. These treatments should not bias our analysis because modification still allows the focal bird to occur both on the archipelago and the continent as priors

**Sensitivity analysis for location estimates of migration**

Different five gamma distribution were tested as movement priors in each run; (shape, rate) = {(13, 0.3), (11, 0.25), (6, 0.25), (3, 0.07), (1, 0.05)}. Geographic distance on the WGS84 ellipsoid was calculated between each pair of points among different runs. Distance calculation was done for the entire track and a region of interest around the East China Sea (ECS). A gamma distribution was fitted to the distribution of calculated differences for each tag, and its mean and standard deviation were calculated from the fitted distribution. The location estimates were also checked graphically, and there was no significant difference that could change our interpretation among the different runs observed.

**Appendix Tables**

**Table S1.1** A list of DNA sequences used in this study. Specimens sequenced for this study and database sequences are both listed. COI haplotype numbers correspond to the median-joining haplotype network, Figure 2a. Concatenated haplotype numbers correspond to the name of OTUs at the Figure S1.2. Accession numbers of the two mitochondrial genes were obtained from DDBJ international DNA database. Some individuals are listed without accession numbers for mitochondrial genes because they share haplotypes with other individuals for which accession numbers were obtained. Refer to the haplotype # for their haplotypes. Accession numbers for nuclear introns are given to each haplotype of individuals sequenced, thus in a case an individual is heterozygous, two accession numbers are given. Sequences of nuclear introns were not obtained for those without the corresponding accession numbers.

| Sampling Locality or Species | COI haplotype # | Concatenated haplotype # (COI & *cytb*) | Accession number (COI) | Accession number (*cytb*) | Accession number (MB) | Accession number (TGFb2) | Collection date (DD.MM.YYYY) | Collection Number | Reference |
| --- | --- | --- | --- | --- | --- | --- | --- | --- | --- |
| Khabarovski krai, Russia | H1 | CH1 | LC512319 | LC512338 | LC549344  LC549372 | LC549395  LC549422 | 09.06.2003 | AK0453 | This study |
| South Primorye, Russia | H2 | CH2 | LC512320 | LC512339 | LC549345 | LC549396  LC549423 | 27.07.1997 | AK0725 | This study |
| South-West Primorye, Russia | H1 | CH3 | LC512321 | LC512340 | LC549346  LC549373 | LC549397  LC549424 | 04.06.1999 | AK0906 | This study |
| South-West Primorye, Russia | H1 | CH1 | - | - | LC549347  LC549374 | LC549398  LC549425 | 07.06.1999 | AK0907 | This study |
| South-West Primorye, Russia | H4 | CH4 | LC512322 | LC512341 | LC549348  LC549375 | LC549399  LC549426 | 07.06.1999 | AK0910 | This study |
| Tymovsky, Sakhalin, Russia | H1 | CH1 | - | - | LC549349  LC549376 | LC549394  LC549421 | 15.06.2010 | AK1673 | This study |
| Nogliky, Sakhalin, Russia | H3 | CH5 | LC512323 | LC512342 | - | - | 09.06.2010 | AK1674 | This study |
| Dunhua, Liaoning Province, China | H5 | CH6 | EF621580 | EF621604 | - | - | - | - | Zhang et al. 2007 |
| Hokkaido, Japan | H8 | CH7 | LC512324 | LC512343 | - | - | 28.5.2017 | LC17-01 | This study |
| Hokkaido, Japan | H8 | CH7 | - | - | LC549352  LC549379 | LC549402 | 28.5.2017 | LC17-02 | This study |
| Hokkaido, Japan | H8 | CH7 | - | - | - | - | 31.5.2017 | LC17-03 | This study |
| Hokkaido, Japan | H6 | CH8 | LC512325 | LC512344 | - | - | 3.6.2017 | LC17-04 | This study |
| Hokkaido, Japan | H8 | CH7 | - | - | - | - | 9.6.2017 | LC17-05 | This study |
| Hokkaido, Japan | H6 | CH10 | - | - | - | - | 16.6.2017 | LC17-06 | This study |
| Hokkaido, Japan | H6 | CH9 | LC512326 | LC512345 | - | - | 21.6.2017 | LC17-07 | This study |
| Hokkaido, Japan | H8 | CH7 | - | - | - | - | 21.6.2017 | LC17-08 | This study |
| Hokkaido, Japan | H8 | CH7 | - | - | LC549354  LC549380 | LC549403 | 21.6.2017 | LC17-09 | This study |
| Hokkaido, Japan | H6 | CH10 | LC512327 | LC512346 | - | - | 26.6.2017 | LC17-11 | This study |
| Hokkaido, Japan | H8 | CH7 | - | - | - | - | 28.6.2017 | LC17-12 | This study |
| Hokkaido, Japan | H7 | CH11 | LC512328 | LC512347 | - | - | 29.6.2017 | LC17-13 | This study |
| Hokkaido, Japan | H7 | CH11 | - | - | - | - | 30.6.2017 | LC17-14 | This study |
| Hokkaido, Japan | H8 | CH7 | - | - | LC549355  LC549381 | LC549404  LC549429 | 1.7.2017 | LC17-15 | This study |
| Hokkaido, Japan | H7 | CH11 | - | - | LC549356  LC549382 | LC549405 | 2.7.2017 | LC17-16 | This study |
| Hokkaido, Japan | H7 | CH11 | - | - | - | - | 3.7.2017 | LC17-17 | This study |
| Hokkaido, Japan | H6 | CH10 | - | - | - | - | 3.7.2017 | LC17-18 | This study |
| Hokkaido, Japan | H6 | CH12 | LC512329 | LC512348 | - | - | 3.7.2017 | LC17-19 | This study |
| Hokkaido, Japan | H8 | CH7 | - | - | - | - | 3.7.2017 | LC17-20 | This study |
| Hokkaido, Japan | H8 | CH7 | - | - | - | - | 3.7.2017 | LC17-21 | This study |
| Hokkaido, Japan | H6 | CH10 | - | - | - | - | 3.7.2017 | LC17-22 | This study |
| Hokkaido, Japan | H6 | CH10 | - | - | - | - | 5.7.2017 | LC17-23 | This study |
| Hokkaido, Japan | H9 | CH13 | LC512330 | LC512349 | LC549357  LC549383 | LC549406  LC549430 | 5.7.2017 | LC17-24 | This study |
| Hokkaido, Japan | H7 | CH11 | - | - | - | - | 5.7.2017 | LC17-25 | This study |
| Hokkaido, Japan | H6 | CH9 | - | - | - | - | 5.7.2017 | LC17-26 | This study |
| Hokkaido, Japan | H8 | CH7 | - | - | - | - | 5.7.2017 | LC17-27 | This study |
| Hokkaido, Japan | H6 | CH10 | - | - | - | - | 6.7.2017 | LC17-28 | This study |
| Hokkaido, Japan | H7 | CH11 | - | - | - | - | 6.7.2017 | LC17-29 | This study |
| Hokkaido, Japan | H8 | CH7 | - | - | - | - | 6.7.2017 | LC17-30 | This study |
| Hokkaido, Japan | H9 | CH13 | - | - | - | - | 9.7.2017 | LC17-31 | This study |
| Hokkaido, Japan | H8 | CH7 | - | - | LC549358  LC549384 | LC549407  LC549431 | 9.7.2017 | LC17-32 | This study |
| Hokkaido, Japan | H7 | CH11 | - | - | - | - | 10.7.2017 | LC17-33 | This study |
| Hokkaido, Japan | H7 | CH11 | - | - | - | - | 10.7.2017 | LC17-34 | This study |
| Hokkaido, Japan | H6 | CH10 | - | - | - | - | 12.7.2017 | LC17-35 | This study |
| Hokkaido, Japan | H8 | CH7 | - | - | - | - | 12.7.2017 | LC17-36 | This study |
| Hokkaido, Japan | H7 | CH11 | - | - | - | - | 13.7.2017 | LC17-37 | This study |
| Hokkaido, Japan | H6 | CH10 | - | - | - | - | 13.7.2017 | LC17-38 | This study |
| Hokkaido, Japan | H6 | CH10 | - | - | - | - | 14.7.2017 | LC17-39 | This study |
| Hokkaido, Japan | H8 | CH7 | - | - | - | - | 14.7.2017 | LC17-40 | This study |
| Hokkaido, Japan | H6 | CH9 | - | - | - | - | 16.7.2017 | LC17-41 | This study |
| Hokkaido, Japan | H7 | CH11 | - | - | - | - | 17.7.2017 | LC17-42 | This study |
| Hokkaido, Japan | H8 | CH7 | - | - | - | - | 19.7.2017 | LC17-43 | This study |
| Hokkaido, Japan | H7 | CH14 | LC512331 | LC512350 | - | - | 19.7.2017 | LC17-44 | This study |
| Hokkaido, Japan | H7 | CH11 | - | - | - | - | 25.7.2017 | LC17-45 | This study |
| Hokkaido, Japan | H8 | CH7 | - | - | - | - | 26.7.2017 | LC17-46 | This study |
| Hokkaido, Japan | H7 | CH11 | - | - | - | - | 28.7.2017 | LC17-47 | This study |
| Hokkaido, Japan | H7 | CH11 | - | - | - | - | 28.7.2017 | LC17-48 | This study |
| Hokkaido, Japan | H8 | CH7 | - | - | - | - | 5.8.2017 | LC17-49 | This study |
| Hokkaido, Japan | H6 | CH10 | - | - | - | - | 5.8.2017 | LC17-50 | This study |
| Hokkaido, Japan | H10 | CH15 | LC512332 | LC512351 | LC549359  LC549385 | LC549408 | 11.8.2017 | LC17-51 | This study |
| Hokkaido, Japan | H10 | CH15 | - | - | - | - | 11.8.2017 | LC17-52 | This study |
| Hokkaido, Japan | H6 | CH10 | - | - | - | - | 12.8.2017 | LC17-53 | This study |
| Hokkaido, Japan | H6 | CH12 | - | - | - | - | 28.5.2018 | LC18-01 | This study |
| Hokkaido, Japan | H8 | CH7 | - | - | - | - | 28.5.2018 | LC18-02 | This study |
| Hokkaido, Japan | H8 | CH7 | - | - | - | - | 29.5.2018 | LC18-03 | This study |
| Hokkaido, Japan | H8 | CH7 | - | - | LC549360 | LC549409  LC549432 | 5.6.2018 | LC18-04 | This study |
| Hokkaido, Japan | H6 | CH10 | - | - | - | - | 5.6.2018 | LC18-05 | This study |
| Hokkaido, Japan | H9 | CH13 | - | - | - | - | 5.6.2018 | LC18-06 | This study |
| Hokkaido, Japan | H6 | CH9 | - | - | - | - | 15.6.2018 | LC18-07 | This study |
| Hokkaido, Japan | H7 | CH11 | - | - | LC549361 | LC549410  LC549433 | 25.7.2018 | LC18-08 | This study |
| Hokkaido, Japan | H6 | CH9 | - | - | LC549362  LC549386 | LC549411  LC549434 | 2.8.2018 | LC18-09 | This study |
| Hokkaido, Japan | H7 | CH11 | - | - | LC549363 | LC549412  LC549435 | 2.8.2018 | LC18-10 | This study |
| Nagano, Japan | H6 | CH10 | - | - | - | - | 30.5.2018 | NC18-01 | This study |
| Nagano, Japan | H7 | CH11 | - | - | - | - | 30.5.2018 | NC18-02 | This study |
| Nagano, Japan | H7 | CH16 | LC512333 | LC512352 | LC549364  LC549387 | LC549413  LC549436 | 30.5.2018 | NC18-03 | This study |
| Nagano, Japan | H6 | CH10 | - | - | - | - | 30.5.2018 | NC18-04 | This study |
| Nagano, Japan | H7 | CH11 | - | - | LC549365 | LC549414  LC549437 | 30.5.2018 | NC18-05 | This study |
| Nagano, Japan | H7 | CH11 | - | - | - | - | 30.5.2018 | NC18-06 | This study |
| Nagano, Japan | H7 | CH17 | LC512334 | LC512353 | - | - | 1.6.2018 | NC18-07 | This study |
| Nagano, Japan | H7 | CH16 | - | - | - | - | 1.6.2018 | NC18-08 | This study |
| Nagano, Japan | H6 | CH10 | - | - | LC549366 | LC549415  LC549438 | 2.6.2018 | NC18-09 | This study |
| Nagano, Japan | H8 | CH7 | - | - | LC549367  LC549388 | LC549416  LC549439 | 2.6.2018 | NC18-10 | This study |
| Nagano, Japan | H7 | CH11 | - | - | - | - | 2.6.2018 | NC18-11 | This study |
| Nagano, Japan | H7 | CH11 | - | - | - | - | 4.7.2018 | NC18-12 | This study |
| Nagano, Japan | H6 | CH10 | - | - | LC549368  LC549389 | LC549417  LC549440 | 4.7.2018 | NC18-13 | This study |
| Nagano, Japan | H8 | CH7 | - | - | - | - | 4.7.2018 | NC18-14 | This study |
| Nagano, Japan | H7 | CH11 | - | - | - | - | 9.7.2018 | NC18-34 | This study |
| Nagano, Japan | H7 | CH11 | - | - | LC549369  LC549390 | LC549418  LC549441 | 7.7.2018 | NC18-35 | This study |
| Nagano, Japan | H7 | CH16 | - | - | - | - | 12.7.2018 | NC18-36 | This study |
| Nagano, Japan | H7 | CH11 | - | - | - | - | 21.6.1997 | 2006-5474 | This study |
| Nagano, Japan | H6 | CH10 | - | - | LC549370 | LC549419 | 27.6.1997 | 1994-0055 | This study |
| Nagano, Japan | H7 | CH18 | LC512335 | LC512354 | - | - | 27.6.1991 | 1994-0054 | This study |
| Hokkaido, Japan | H6 | CH10 | - | - | - | - | 17.6.1998 | 3C-17533 | This study |
| Hokkaido, Japan | H7 | CH11 | - | - | - | - | 17.6.1998 | 3C-17534 | This study |
| Hokkaido, Japan | H6 | CH9 | - | - | - | - | 17.6.1998 | 3C-17535 | This study |
| Ryukyu, Japan | H11 | CH20 | LC512337 | LC512356 | LC549351  LC549378 | LC549401  LC549428 | 8.5.2010 | 2010-0363 | This study |
| Ryukyu, Japan | H11 | CH19 | LC512336 | LC512355 | LC549350  LC549377 | LC549400  LC549427 | 15.9.1997 | 1997-0359 | This study |
| Shanbei, Shaanxi Province, China | H11 | CH21 | EF621579 | EF621603 | - | - | - | - | Zhang et al. 2007 |
| Xi’an, Shaanxi Province, China | H11 | CH22 | EF621578 | EF621602 | - | - | - | - | Zhang et al. 2007 |
| Ryukyu, Japan | H11 | - | - | - | - | - | 28.8.2007 | BJNSM613-10 | BOLDsystem |
| Calabarzon, Philippines | H11 | - | - | - | - | - | 24.2.2010 | BUPD010-11 | BOLDsystem |
| Calabarzon, Philippines | H11 | - | - | - | - | - | 4.3.2010 | BUPD011-11 | BOLDsystem |
| Luzon, Philippines | H12 | - | - | - | - | - | 27.4.1989 | USNMA183-10 | BOLDsystem |
| Calabarzon, Philippines | H13 | - | - | - | - | - | 19.1.2010 | BUPD009-11 | BOLDsystem |
| Calabarzon, Philippines | H14 | - | - | - | - | - | 22.4.2010 | BUPD013-11 | BOLDsystem |
| Calabarzon, Philippines | H1 | - | - | - | - | - | 21.4.2010 | BUPD012-11 | BOLDsystem |
| Khabarovskiy Kray, Russia | H1 | - | - | - | - | - | 13.6.1993 | KBPBU615-06 | BOLDsystem |
| Magadanskaya Oblast, Russia | H1 | - | - | - | - | - | 1.9.1992 | KBPBU616-06 | BOLDsystem |
| Tyva Republic, Russia | H1 | - | - | - | - | - | 4.6.1994 | KBPBU617-06 | BOLDsystem |
| Outgroup | | | | | | | | | |
| *Lanius bucephalus* (China) | - | - | EF621577 | EF621601 | - | - | - | - | Zhang et al. 2007 |
| *Lanius bucephalus* (Japan) | HLb | CHLb | LC550389 | LC550390 | LC549341  LC549371 | LC549391 | 5.2013 | ESLB13-06 | This study |
| *Lanius bucephalus* (Japan) | HLb | CHLb | - | - | LC549342 | LC549392 | 5.2013 | ESLB13-08 | This study |
| *Lanius bucephalus* (Japan) | HLb | CHLb | - | - | LC549343 | LC549393  LC549420 | 5.2013 | ESLB13-09 | This study |
| *Lanius tephronotus* (multiple individual) | - | - | EF621573 | EF621597 | HQ996794 | HQ996888 | - | - | Zhang et al. 2007  Fuchs et al. 2011 |
| *Lanius validirostris*  (multiple individual) | - | - | - | - | HQ996789 | HQ996884 |  | - | Fuchs et al. 2011 |
| *Lanius schach* | - | - | EF621576 | EF621600 | - | - | - | - | Zhang et al. 2007 |
| *Lanius isabellinus (isabellinus/phoenicuroides)* | - | - | - | - | EF635049  EF635050 | HQ996887 | - | - | Gonzalez et al. 2008  Fuchs et al. 2011 |
| *Lanius collurio*  (multiple individual) | - | - | - | - | AY228328  EF635051  EF635052 | KU719017 HQ996886 HQ996885  KU719021  KU719020  KU719019  KU719018  KU719015  KU719014  KU719013  KU719012  KU719011  KU719010  KU719007  KU719006  KU719005  KU719004 | - | - | Ericson & Johansson 2003  Gonzalez et al. 2008  Hung et al. 2016  Fuchs et al. 2011 |
| *Lanius tigrinus* | - | - | - | - | MF458414 | - | - | - | Hooper & Price 2017 |

**Table S1.2** Results from the sensitivity analyses of location estimates in migratory analysis. Mean difference of distances between every pair of points and its standard deviation for the entire track and a region of interest around the East China Sea (ECS) are shown for each geolocator tag.

| **Tag #** | **Mean distance for the entire track (SD) [km]** | **Mean distance for the track around the ECS (SD) [km]** |
| --- | --- | --- |
| **V5604-021** | 86.5 (113.5) | 48.0 (50.4) |
| **V5604-025** | 46.7 (50.6) | 49.9 (52.5) |
| **V5604-029** | 68.0 (84.0) | 58.3 (67.6) |

**Captions for Appendix Figures**

**Figure S1.1** Final occurrence records used for the present-only species distribution modelling (green coloured dots).

**Figure S1.2** A Bayesian inference phylogenetic tree reconstructed using 1,789 bp of mitochondrial DNA, presenting concatenated *cytb* and COI genes. Grey bars indicate 95% highest posterior densities for divergence time estimates. Numbers on nodes indicate their posterior probabilities.

**Figure S1.3** Projection of the species distribution model (SDM) constructed for *Lanius cristatus cristatus* to the three different climate models of the last glacial maximum (LGM). Refer Figure 3 in the main text for details on the graphical presentation. Note that higher glacial suitability was estimated around the present Korean Peninsula and northern China in every climate model.

**Figure S1.4** Projection of the species distribution model (SDM) constructed for *Lanius cristatus lucionensis* to the three different climate models of the last glacial maximum (LGM). Refer Figure 3 in the main text for details on the graphical presentation. Note that higher glacial suitability was estimated around the present Korean Peninsula and northern China in every climate model.

**Figure S1.5** Location estimates of entire migration tracks for the three individuals. Red and white dots indicate stop-over sites and migrating paths, respectively. Relative time spent for stationary period (breeding, stop-over and wintering) was indicated as posterior probability distribution for each individual in colour gradation, increasing from yellow, green to indigo.

Reference:

Ericson, P. G. P., & Johansson, U. S. (2003). Phylogeography of Passerida (Aves: Passeriformes) based on nuclear and mitochondrial sequence data. Molecular Phylogenetics and Evolution, 29(1), 126–138.

Fuchs, J., Crowe, T. M., & Bowie, R. C. K. (2011). Phylogeography of the fiscal shrike (*Lanius collaris*): a novel pattern of genetic structure across the arid zones and savannas of Africa. Journal of Biogeography, 38(11), 2210–2222.

Gonzalez, J., Wink, M., Garcia-del-Rey, E., & Castro, G. D. (2008). Evidence from DNA nucleotide sequences and ISSR profiles indicate paraphyly in subspecies of the Southern Grey Shrike (*Lanius meridionalis*). Journal of Ornithology, 149(4), 495–506.

Heslewood M. M., Elphinstone, M. S., Tidemann, S. C., Baverstock, P. R. (1998). Myoglobin intron variation in the Gouldian Finch *Erythrura gouldiae* assessed by temperature gradient gel electrophoresis. Electrophoresis, 19, 142–151.

Hijmans, R. J., Phillips, S., Leathwick, J., & Elith, J. (2013). Dismo: Species distribution modelling.

Hooper, D. M., & Price, T. D. (2017). Chromosomal inversion differences correlate with range overlap in passerine birds. Nature Ecology and Evolution, 1(10), 1526-1534.

Hung, C.-M., Drovetski, S. V., & Zink, R. M. (2016). Matching loci surveyd to questions asked in phylogeography. Proceedings of the Royal Society B. 283, 20152340.

Imamura, K. (1976). Breeding of Lanius cristatus lucionensis in Kyushu. *Tori*, *99*(25), 53–56.

Kitazawa, M., Senzaki, M., Matsumiya, H., Hara, S., & Mizumura, H. (2020). Drastic decline in the endemic brown shrike subspecies *Lanius cristatus superciliosus* in Japan. Bird Conservation International, doi:10.1017/S0959270920000556.

Lefranc, N., & Worfolk, T. (1997). *Shrikes: a guide to the shrikes of the world*. East Sussex: Pica Press.

Primmer, C. R., Borge, T., Lindell, J., & Sætre, G.-P. (2002). Single-nucleotide polymorphism characterization in species with limited availble sequence information: high nucleotide diversity revealed in the avian genome. Molecular Ecology, 11, 603–612.

Slade, R. W., Moritz, C., Heideman, A., & Hale, P. T. (1993). Rapid assessment of single-copy nuclear DNA variation in diverse species. Molecular Ecology, 2, 359–373.

The Ornithological Society of Japan. (2012). *Check-list of Japanese Birds, 7th revised edition*. Sanda: Ornithological Society of Japan.

Untergasser, A., Nijveen, H., Rao, X., & Bisseling, T. (2017). Primer3Plus , an enhanced web interface to Primer3, *35*, 71–74.

Zhang, W., Lei, F. M., Liang, G., Yin, Z. H. Zhao, H. F. Wang, H. J., & Krištín, A. (2007). Taxonomic status of eight Asian shrike species (*Lanius*): Phylogenetic analysis based on Cyt *b* and CoI gene sequences. 42(2), 173–180.

Zizka, A., Silvestro, D., Andermann, T., Azevedo, J., Ritter, C. D., Edler, D., Farooq, H., Herdean, A., Ariza, M., Scharn, R., Svantesson, S., Wengström, N., Zizka, V., & Antonelli, A. (2019). CooridnateCleaner: Standardized cleaning of occurrence records biological collection databases. Methods in Ecology and Evolution, 10, 744–751.
