# Supplementary material for: Migration‐tracking integrated phylogeography supports long‐distance dispersal‐driven divergence for a migratory bird species in the Japanese archipelago
Source: Ecol Evol. 2021 May 2;11(11):6066–79. doi: 10.1002/ece3.7387 (PMC8207368; doi:10.1002/ece3.7387)
Supplement: Supplementary file 7 — Table S1.1‐S1.2 [file ECE3-11-6066-s006.docx]

**Appendix Tables**

**Table S1.1** A list of DNA sequences used in this study. Specimens sequenced for this study and database sequences are both listed. COI haplotype numbers correspond to the median-joining haplotype network, Figure 2a. Concatenated haplotype numbers correspond to the name of OTUs at the Figure S1.2. Accession numbers of the two mitochondrial genes were obtained from DDBJ international DNA database. Some individuals are listed without accession numbers for mitochondrial genes because they share haplotypes with other individuals for which accession numbers were obtained. Refer to the haplotype # for their haplotypes. Accession numbers for nuclear introns are given to each haplotype of individuals sequenced, thus in a case an individual is heterozygous, two accession numbers are given. Sequences of nuclear introns were not obtained for those without the corresponding accession numbers.

| Sampling Locality or Species | COI haplotype # | Concatenated haplotype # (COI & *cytb*) | Accession number (COI) | Accession number (*cytb*) | Accession number (MB) | Accession number (TGFb2) | Collection date (DD.MM.YYYY) | Collection Number | Reference |
| --- | --- | --- | --- | --- | --- | --- | --- | --- | --- |
| Khabarovski krai, Russia | H1 | CH1 | LC512319 | LC512338 | LC549344  LC549372 | LC549395  LC549422 | 09.06.2003 | AK0453 | This study |
| South Primorye, Russia | H2 | CH2 | LC512320 | LC512339 | LC549345 | LC549396  LC549423 | 27.07.1997 | AK0725 | This study |
| South-West Primorye, Russia | H1 | CH3 | LC512321 | LC512340 | LC549346  LC549373 | LC549397  LC549424 | 04.06.1999 | AK0906 | This study |
| South-West Primorye, Russia | H1 | CH1 | - | - | LC549347  LC549374 | LC549398  LC549425 | 07.06.1999 | AK0907 | This study |
| South-West Primorye, Russia | H4 | CH4 | LC512322 | LC512341 | LC549348  LC549375 | LC549399  LC549426 | 07.06.1999 | AK0910 | This study |
| Tymovsky, Sakhalin, Russia | H1 | CH1 | - | - | LC549349  LC549376 | LC549394  LC549421 | 15.06.2010 | AK1673 | This study |
| Nogliky, Sakhalin, Russia | H3 | CH5 | LC512323 | LC512342 | - | - | 09.06.2010 | AK1674 | This study |
| Dunhua, Liaoning Province, China | H5 | CH6 | EF621580 | EF621604 | - | - | - | - | Zhang et al. 2007 |
| Hokkaido, Japan | H8 | CH7 | LC512324 | LC512343 | - | - | 28.5.2017 | LC17-01 | This study |
| Hokkaido, Japan | H8 | CH7 | - | - | LC549352  LC549379 | LC549402 | 28.5.2017 | LC17-02 | This study |
| Hokkaido, Japan | H8 | CH7 | - | - | - | - | 31.5.2017 | LC17-03 | This study |
| Hokkaido, Japan | H6 | CH8 | LC512325 | LC512344 | - | - | 3.6.2017 | LC17-04 | This study |
| Hokkaido, Japan | H8 | CH7 | - | - | - | - | 9.6.2017 | LC17-05 | This study |
| Hokkaido, Japan | H6 | CH10 | - | - | - | - | 16.6.2017 | LC17-06 | This study |
| Hokkaido, Japan | H6 | CH9 | LC512326 | LC512345 | - | - | 21.6.2017 | LC17-07 | This study |
| Hokkaido, Japan | H8 | CH7 | - | - | - | - | 21.6.2017 | LC17-08 | This study |
| Hokkaido, Japan | H8 | CH7 | - | - | LC549354  LC549380 | LC549403 | 21.6.2017 | LC17-09 | This study |
| Hokkaido, Japan | H6 | CH10 | LC512327 | LC512346 | - | - | 26.6.2017 | LC17-11 | This study |
| Hokkaido, Japan | H8 | CH7 | - | - | - | - | 28.6.2017 | LC17-12 | This study |
| Hokkaido, Japan | H7 | CH11 | LC512328 | LC512347 | - | - | 29.6.2017 | LC17-13 | This study |
| Hokkaido, Japan | H7 | CH11 | - | - | - | - | 30.6.2017 | LC17-14 | This study |
| Hokkaido, Japan | H8 | CH7 | - | - | LC549355  LC549381 | LC549404  LC549429 | 1.7.2017 | LC17-15 | This study |
| Hokkaido, Japan | H7 | CH11 | - | - | LC549356  LC549382 | LC549405 | 2.7.2017 | LC17-16 | This study |
| Hokkaido, Japan | H7 | CH11 | - | - | - | - | 3.7.2017 | LC17-17 | This study |
| Hokkaido, Japan | H6 | CH10 | - | - | - | - | 3.7.2017 | LC17-18 | This study |
| Hokkaido, Japan | H6 | CH12 | LC512329 | LC512348 | - | - | 3.7.2017 | LC17-19 | This study |
| Hokkaido, Japan | H8 | CH7 | - | - | - | - | 3.7.2017 | LC17-20 | This study |
| Hokkaido, Japan | H8 | CH7 | - | - | - | - | 3.7.2017 | LC17-21 | This study |
| Hokkaido, Japan | H6 | CH10 | - | - | - | - | 3.7.2017 | LC17-22 | This study |
| Hokkaido, Japan | H6 | CH10 | - | - | - | - | 5.7.2017 | LC17-23 | This study |
| Hokkaido, Japan | H9 | CH13 | LC512330 | LC512349 | LC549357  LC549383 | LC549406  LC549430 | 5.7.2017 | LC17-24 | This study |
| Hokkaido, Japan | H7 | CH11 | - | - | - | - | 5.7.2017 | LC17-25 | This study |
| Hokkaido, Japan | H6 | CH9 | - | - | - | - | 5.7.2017 | LC17-26 | This study |
| Hokkaido, Japan | H8 | CH7 | - | - | - | - | 5.7.2017 | LC17-27 | This study |
| Hokkaido, Japan | H6 | CH10 | - | - | - | - | 6.7.2017 | LC17-28 | This study |
| Hokkaido, Japan | H7 | CH11 | - | - | - | - | 6.7.2017 | LC17-29 | This study |
| Hokkaido, Japan | H8 | CH7 | - | - | - | - | 6.7.2017 | LC17-30 | This study |
| Hokkaido, Japan | H9 | CH13 | - | - | - | - | 9.7.2017 | LC17-31 | This study |
| Hokkaido, Japan | H8 | CH7 | - | - | LC549358  LC549384 | LC549407  LC549431 | 9.7.2017 | LC17-32 | This study |
| Hokkaido, Japan | H7 | CH11 | - | - | - | - | 10.7.2017 | LC17-33 | This study |
| Hokkaido, Japan | H7 | CH11 | - | - | - | - | 10.7.2017 | LC17-34 | This study |
| Hokkaido, Japan | H6 | CH10 | - | - | - | - | 12.7.2017 | LC17-35 | This study |
| Hokkaido, Japan | H8 | CH7 | - | - | - | - | 12.7.2017 | LC17-36 | This study |
| Hokkaido, Japan | H7 | CH11 | - | - | - | - | 13.7.2017 | LC17-37 | This study |
| Hokkaido, Japan | H6 | CH10 | - | - | - | - | 13.7.2017 | LC17-38 | This study |
| Hokkaido, Japan | H6 | CH10 | - | - | - | - | 14.7.2017 | LC17-39 | This study |
| Hokkaido, Japan | H8 | CH7 | - | - | - | - | 14.7.2017 | LC17-40 | This study |
| Hokkaido, Japan | H6 | CH9 | - | - | - | - | 16.7.2017 | LC17-41 | This study |
| Hokkaido, Japan | H7 | CH11 | - | - | - | - | 17.7.2017 | LC17-42 | This study |
| Hokkaido, Japan | H8 | CH7 | - | - | - | - | 19.7.2017 | LC17-43 | This study |
| Hokkaido, Japan | H7 | CH14 | LC512331 | LC512350 | - | - | 19.7.2017 | LC17-44 | This study |
| Hokkaido, Japan | H7 | CH11 | - | - | - | - | 25.7.2017 | LC17-45 | This study |
| Hokkaido, Japan | H8 | CH7 | - | - | - | - | 26.7.2017 | LC17-46 | This study |
| Hokkaido, Japan | H7 | CH11 | - | - | - | - | 28.7.2017 | LC17-47 | This study |
| Hokkaido, Japan | H7 | CH11 | - | - | - | - | 28.7.2017 | LC17-48 | This study |
| Hokkaido, Japan | H8 | CH7 | - | - | - | - | 5.8.2017 | LC17-49 | This study |
| Hokkaido, Japan | H6 | CH10 | - | - | - | - | 5.8.2017 | LC17-50 | This study |
| Hokkaido, Japan | H10 | CH15 | LC512332 | LC512351 | LC549359  LC549385 | LC549408 | 11.8.2017 | LC17-51 | This study |
| Hokkaido, Japan | H10 | CH15 | - | - | - | - | 11.8.2017 | LC17-52 | This study |
| Hokkaido, Japan | H6 | CH10 | - | - | - | - | 12.8.2017 | LC17-53 | This study |
| Hokkaido, Japan | H6 | CH12 | - | - | - | - | 28.5.2018 | LC18-01 | This study |
| Hokkaido, Japan | H8 | CH7 | - | - | - | - | 28.5.2018 | LC18-02 | This study |
| Hokkaido, Japan | H8 | CH7 | - | - | - | - | 29.5.2018 | LC18-03 | This study |
| Hokkaido, Japan | H8 | CH7 | - | - | LC549360 | LC549409  LC549432 | 5.6.2018 | LC18-04 | This study |
| Hokkaido, Japan | H6 | CH10 | - | - | - | - | 5.6.2018 | LC18-05 | This study |
| Hokkaido, Japan | H9 | CH13 | - | - | - | - | 5.6.2018 | LC18-06 | This study |
| Hokkaido, Japan | H6 | CH9 | - | - | - | - | 15.6.2018 | LC18-07 | This study |
| Hokkaido, Japan | H7 | CH11 | - | - | LC549361 | LC549410  LC549433 | 25.7.2018 | LC18-08 | This study |
| Hokkaido, Japan | H6 | CH9 | - | - | LC549362  LC549386 | LC549411  LC549434 | 2.8.2018 | LC18-09 | This study |
| Hokkaido, Japan | H7 | CH11 | - | - | LC549363 | LC549412  LC549435 | 2.8.2018 | LC18-10 | This study |
| Nagano, Japan | H6 | CH10 | - | - | - | - | 30.5.2018 | NC18-01 | This study |
| Nagano, Japan | H7 | CH11 | - | - | - | - | 30.5.2018 | NC18-02 | This study |
| Nagano, Japan | H7 | CH16 | LC512333 | LC512352 | LC549364  LC549387 | LC549413  LC549436 | 30.5.2018 | NC18-03 | This study |
| Nagano, Japan | H6 | CH10 | - | - | - | - | 30.5.2018 | NC18-04 | This study |
| Nagano, Japan | H7 | CH11 | - | - | LC549365 | LC549414  LC549437 | 30.5.2018 | NC18-05 | This study |
| Nagano, Japan | H7 | CH11 | - | - | - | - | 30.5.2018 | NC18-06 | This study |
| Nagano, Japan | H7 | CH17 | LC512334 | LC512353 | - | - | 1.6.2018 | NC18-07 | This study |
| Nagano, Japan | H7 | CH16 | - | - | - | - | 1.6.2018 | NC18-08 | This study |
| Nagano, Japan | H6 | CH10 | - | - | LC549366 | LC549415  LC549438 | 2.6.2018 | NC18-09 | This study |
| Nagano, Japan | H8 | CH7 | - | - | LC549367  LC549388 | LC549416  LC549439 | 2.6.2018 | NC18-10 | This study |
| Nagano, Japan | H7 | CH11 | - | - | - | - | 2.6.2018 | NC18-11 | This study |
| Nagano, Japan | H7 | CH11 | - | - | - | - | 4.7.2018 | NC18-12 | This study |
| Nagano, Japan | H6 | CH10 | - | - | LC549368  LC549389 | LC549417  LC549440 | 4.7.2018 | NC18-13 | This study |
| Nagano, Japan | H8 | CH7 | - | - | - | - | 4.7.2018 | NC18-14 | This study |
| Nagano, Japan | H7 | CH11 | - | - | - | - | 9.7.2018 | NC18-34 | This study |
| Nagano, Japan | H7 | CH11 | - | - | LC549369  LC549390 | LC549418  LC549441 | 7.7.2018 | NC18-35 | This study |
| Nagano, Japan | H7 | CH16 | - | - | - | - | 12.7.2018 | NC18-36 | This study |
| Nagano, Japan | H7 | CH11 | - | - | - | - | 21.6.1997 | 2006-5474 | This study |
| Nagano, Japan | H6 | CH10 | - | - | LC549370 | LC549419 | 27.6.1997 | 1994-0055 | This study |
| Nagano, Japan | H7 | CH18 | LC512335 | LC512354 | - | - | 27.6.1991 | 1994-0054 | This study |
| Hokkaido, Japan | H6 | CH10 | - | - | - | - | 17.6.1998 | 3C-17533 | This study |
| Hokkaido, Japan | H7 | CH11 | - | - | - | - | 17.6.1998 | 3C-17534 | This study |
| Hokkaido, Japan | H6 | CH9 | - | - | - | - | 17.6.1998 | 3C-17535 | This study |
| Ryukyu, Japan | H11 | CH20 | LC512337 | LC512356 | LC549351  LC549378 | LC549401  LC549428 | 8.5.2010 | 2010-0363 | This study |
| Ryukyu, Japan | H11 | CH19 | LC512336 | LC512355 | LC549350  LC549377 | LC549400  LC549427 | 15.9.1997 | 1997-0359 | This study |
| Shanbei, Shaanxi Province, China | H11 | CH21 | EF621579 | EF621603 | - | - | - | - | Zhang et al. 2007 |
| Xi’an, Shaanxi Province, China | H11 | CH22 | EF621578 | EF621602 | - | - | - | - | Zhang et al. 2007 |
| Ryukyu, Japan | H11 | - | - | - | - | - | 28.8.2007 | BJNSM613-10 | BOLDsystem |
| Calabarzon, Philippines | H11 | - | - | - | - | - | 24.2.2010 | BUPD010-11 | BOLDsystem |
| Calabarzon, Philippines | H11 | - | - | - | - | - | 4.3.2010 | BUPD011-11 | BOLDsystem |
| Luzon, Philippines | H12 | - | - | - | - | - | 27.4.1989 | USNMA183-10 | BOLDsystem |
| Calabarzon, Philippines | H13 | - | - | - | - | - | 19.1.2010 | BUPD009-11 | BOLDsystem |
| Calabarzon, Philippines | H14 | - | - | - | - | - | 22.4.2010 | BUPD013-11 | BOLDsystem |
| Calabarzon, Philippines | H1 | - | - | - | - | - | 21.4.2010 | BUPD012-11 | BOLDsystem |
| Khabarovskiy Kray, Russia | H1 | - | - | - | - | - | 13.6.1993 | KBPBU615-06 | BOLDsystem |
| Magadanskaya Oblast, Russia | H1 | - | - | - | - | - | 1.9.1992 | KBPBU616-06 | BOLDsystem |
| Tyva Republic, Russia | H1 | - | - | - | - | - | 4.6.1994 | KBPBU617-06 | BOLDsystem |
| Outgroup | | | | | | | | | |
| *Lanius bucephalus* (China) | - | - | EF621577 | EF621601 | - | - | - | - | Zhang et al. 2007 |
| *Lanius bucephalus* (Japan) | HLb | CHLb | LC550389 | LC550390 | LC549341  LC549371 | LC549391 | 5.2013 | ESLB13-06 | This study |
| *Lanius bucephalus* (Japan) | HLb | CHLb | - | - | LC549342 | LC549392 | 5.2013 | ESLB13-08 | This study |
| *Lanius bucephalus* (Japan) | HLb | CHLb | - | - | LC549343 | LC549393  LC549420 | 5.2013 | ESLB13-09 | This study |
| *Lanius tephronotus* (multiple individual) | - | - | EF621573 | EF621597 | HQ996794 | HQ996888 | - | - | Zhang et al. 2007  Fuchs et al. 2011 |
| *Lanius validirostris*  (multiple individual) | - | - | - | - | HQ996789 | HQ996884 |  | - | Fuchs et al. 2011 |
| *Lanius schach* | - | - | EF621576 | EF621600 | - | - | - | - | Zhang et al. 2007 |
| *Lanius isabellinus (isabellinus/phoenicuroides)* | - | - | - | - | EF635049  EF635050 | HQ996887 | - | - | Gonzalez et al. 2008  Fuchs et al. 2011 |
| *Lanius collurio*  (multiple individual) | - | - | - | - | AY228328  EF635051  EF635052 | KU719017 HQ996886 HQ996885  KU719021  KU719020  KU719019  KU719018  KU719015  KU719014  KU719013  KU719012  KU719011  KU719010  KU719007  KU719006  KU719005  KU719004 | - | - | Ericson & Johansson 2003  Gonzalez et al. 2008  Hung et al. 2016  Fuchs et al. 2011 |
| *Lanius tigrinus* | - | - | - | - | MF458414 | - | - | - | Hooper & Price 2017 |

**Table S1.2** Results from the sensitivity analyses of location estimates in migratory analysis. Mean difference of distances between every pair of points and its standard deviation for the entire track and a region of interest around the East China Sea (ECS) are shown for each geolocator tag.

| **Tag #** | **Mean distance for the entire track (SD) [km]** | **Mean distance for the track around the ECS (SD) [km]** |
| --- | --- | --- |
| **V5604-021**  **V5604-025**  **V5604-029** | 86.5 (113.5)  46.7 (50.6)  68.0 (84.0) | 48.0 (50.4)  49.9 (52.5)  58.3 (67.6) |
